# ‘Shawl Sign’ as a Paraneoplastic Dermatosis

**DOI:** 10.7759/cureus.7498

**Published:** 2020-04-01

**Authors:** Eukesh Ranjit, Amit Sapra, Airn Etherton, Waiz Wasey, Priyanka Bhandari

**Affiliations:** 1 Family Medicine, Southern Illinois University School of Medicine, Springfield, USA; 2 Family and Community Medicine, Southern Illinois University School of Medicine, Springfield, USA

**Keywords:** skin rash, paraneoplastic syndrome, malignancy, shawl sign, metastatic disease, atypical rash, dermatomyositis, atypical presentation, sunburn, skin biopsy

## Abstract

Dermatomyositis (DM) is an idiopathic condition characterized by inflammation of muscles and skin lesions. It is often a paraneoplastic manifestation of internal malignancy. Hence, early recognition of this disorder is important. Although not all dermatomyositis are associated with malignancies, the ones with such association regress with the treatment of associated malignancy. In clinical practice, symptoms of muscle weakness can be vague, and skin lesions can be dismissed as sunburn. We present a case of an elder adult female who presented with dermatomyositis as a paraneoplastic syndrome secondary to an underlying Mullerian malignancy.

## Introduction

Paraneoplastic syndromes are non-malignant disorders arising from tumor secretion of peptides, hormones, or cytokines or from immune cross-reactivity between malignant and normal tissues. Paraneoplastic syndromes may affect dermatologic, endocrine, neurologic, rheumatologic, and hematologic systems and are commonly associated with gynecologic tumors, small cell lung cancer, breast cancer, and hematologic malignancies [1].

Dermatomyositis can present as a paraneoplastic dermatosis and may present as a characteristic erythematous rash distributed in a “shawl” pattern over the neck, upper back, chest and shoulders.

We present a case of a 69-year-old female who had visited our clinic with a diffuse rash over the upper part of the chest wall and upper back. The patient reported that she has had sunburns in similar locations before, but she had not been out in the sun lately. A series of investigations revealed her condition to be far more complicated and, in fact, related to her underlying malignancy.

## Case presentation

Our patient is a 69-year-old Caucasian female with a past medical history of type II diabetes, hypertension, hyperlipidemia, obesity, obstructive sleep apnea, and seborrheic dermatitis. She presented to the Family Medicine clinic for evaluation of abdominal pain after a fall on the ice at home. She developed symptoms of abdominal discomfort and bloating in an area where she had an abdominal hernia repair five years prior. The hernia repair had been complicated by wound dehiscence and infection that required skin grafting and subsequent surgical repair. Past surgical history also included cholecystectomy and hysterectomy for uterine fibroids. She is a nonsmoker and rarely drinks alcohol.

**Figure 1:**
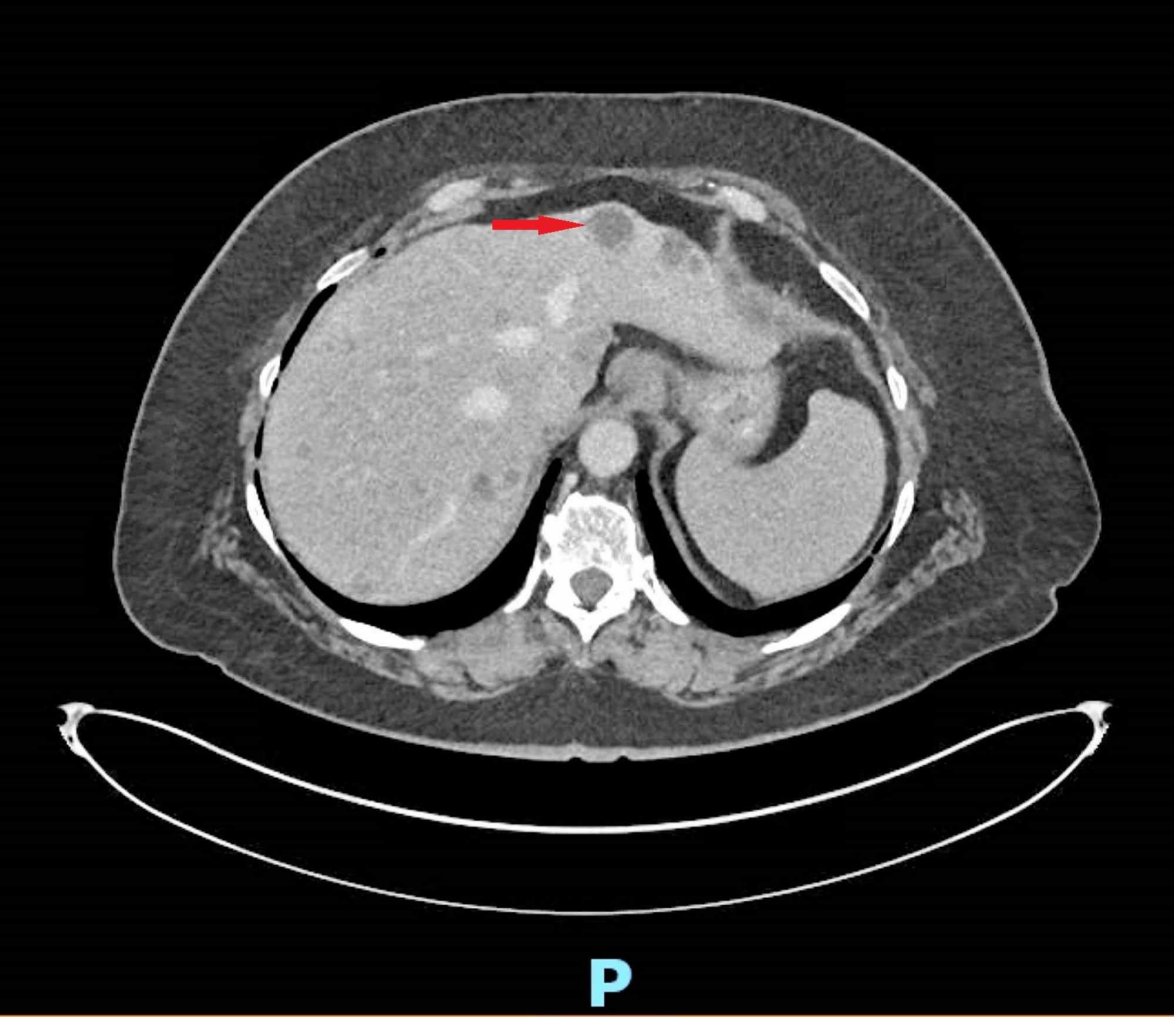
CT scan of the abdomen and pelvis with contrast showing hepatic lesions concerning for metastatic disease.

Considering numerous abdominal surgeries and abdominal pain, a CT scan of her abdomen and pelvis with and without contrast was ordered. CT scan revealed innumerable bilateral hepatic lesions (Figure 1) concerning for metastatic disease along with multiple enlarged lymph nodes in the porta hepatis and several indeterminate right lower lobe pulmonary nodules.

**Figure 2:**
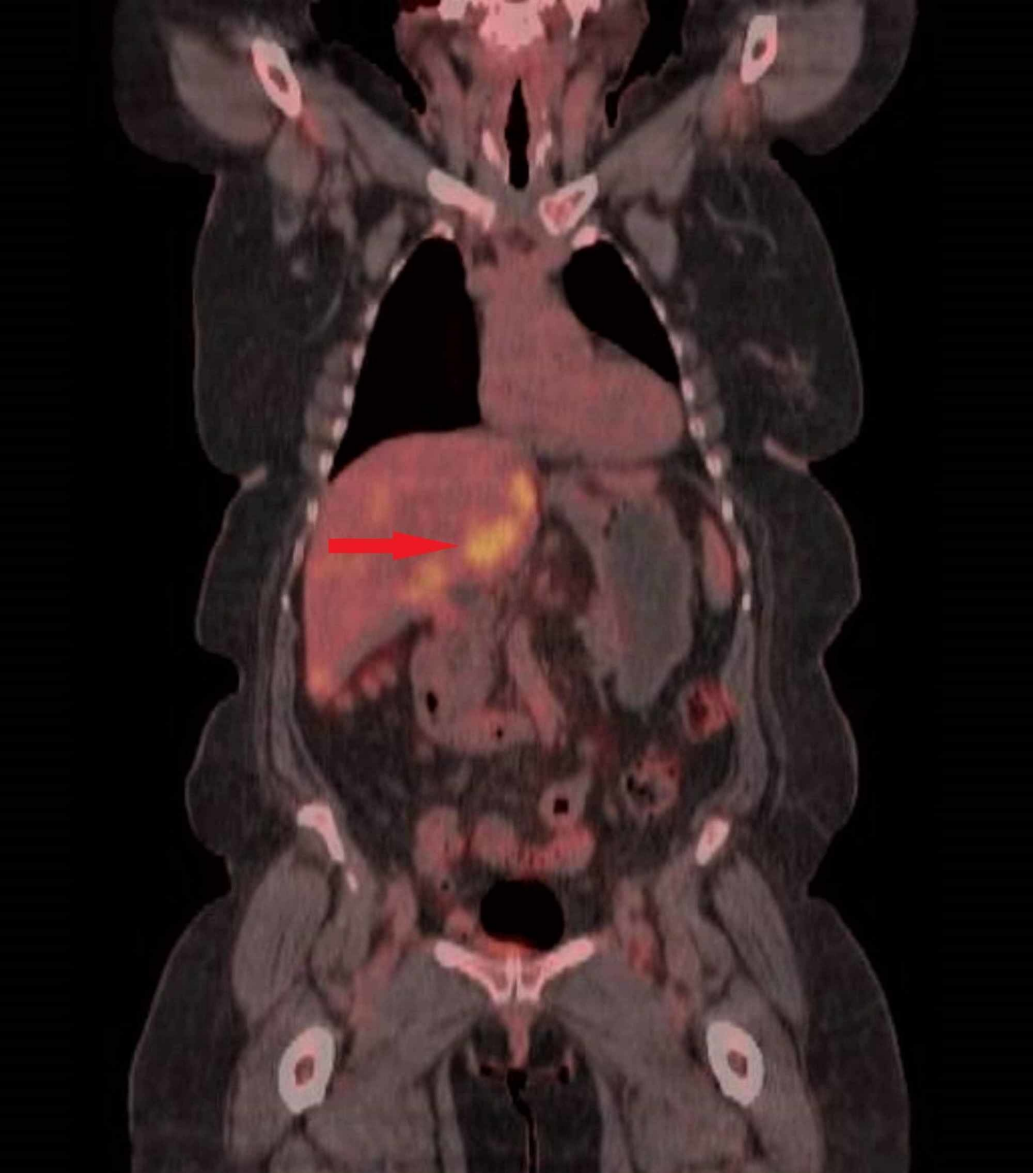
Positron emission tomography (PET) scan showing diffuse hepatic metastatic disease.

A positron emission tomography (PET) scan was then ordered that revealed a hypermetabolic soft tissue lesion in the anterior mediastinum measuring 4 cm, mildly hypermetabolic subpleural nodules were noted in the right lung, and there was also hypermetabolic lymphadenopathy in the porta hepatis. Multiple hypermetabolic hepatic metastases were seen (Figure 2).

Lab work, including complete metabolic panel (CMP), complete blood count (CBC) with differential, lactate dehydrogenase (LDH), carbohydrate antigen 19-9 (CA 19-9), cancer antigen 125 (CA 125), alpha-fetoprotein (AFP), and a pathology slide review was ordered. Her white blood cell (WBC) was elevated at 18.5, with a left shift. Her LDH was elevated at 501, and CA 125 tumor marker was elevated at 1096. Her peripheral smear result revealed absolute neutrophilia, absolute monocytosis, absolute lymphopenia. Other labs were unremarkable, including liver enzymes.

**Figure 3:**
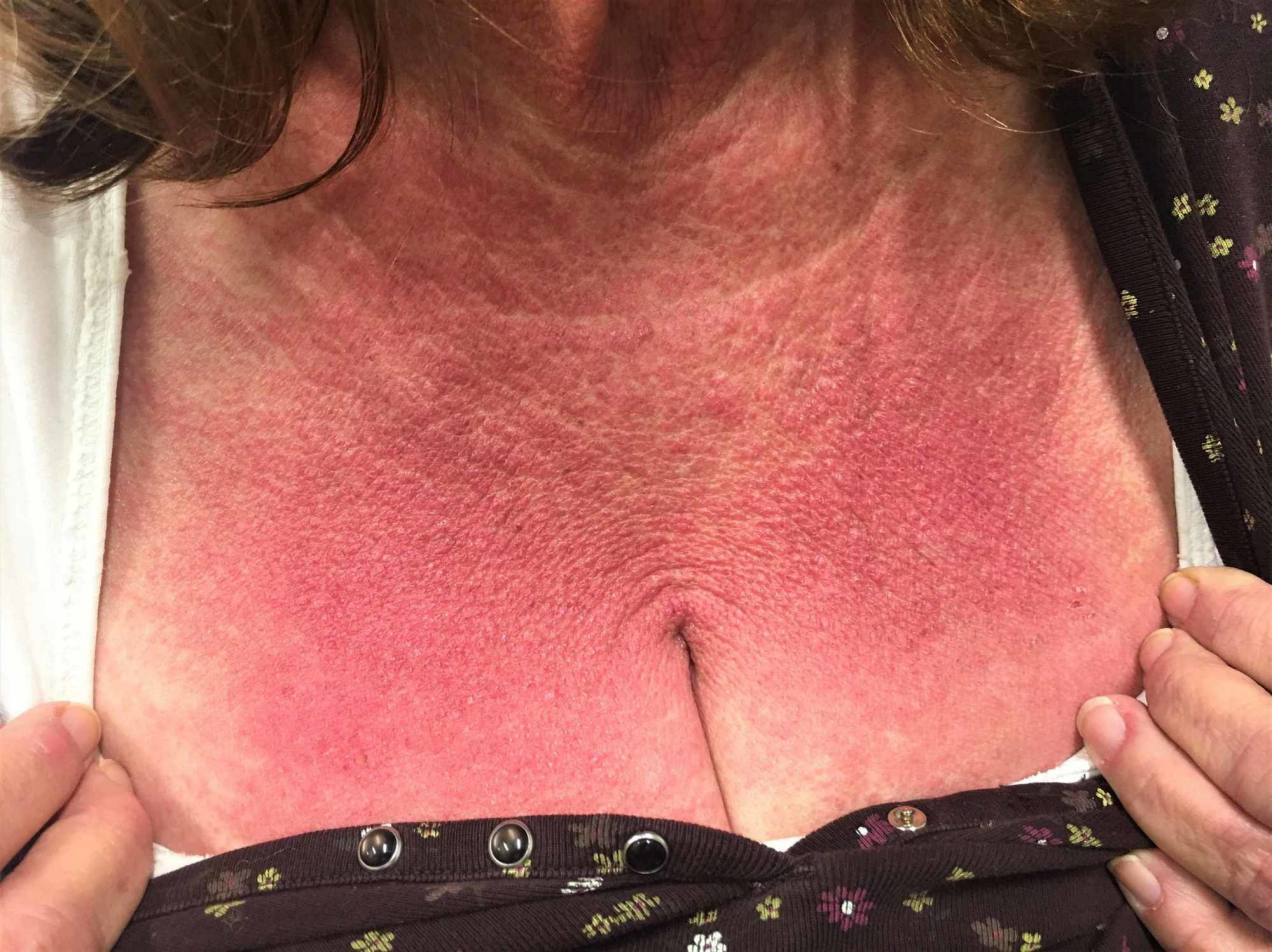
Red, maculopapular well-demarcated rash on the chest wall ‘Shawl sign’.

The patient returned after four weeks to the clinic to review test results and evaluation. At this visit, she reported the development of a new rash over the last 2-3 weeks, which started at the hairline and spread down the neck, upper shoulders, and across her chest and that it was itching and burning. During the exam, she was noted to have a well-demarcated maculopapular rash to her chest wall, forehead, posterior upper back, and upper shoulders. She had attributed it to her seborrheic dermatitis spreading from her scalp region. She denied any recent sun exposure, new medications, or other exposures (clothes, detergent, pets). She had been using clobetasol ointment she had at home with no relief to the rash (Figure 3).

**Figure 4:**
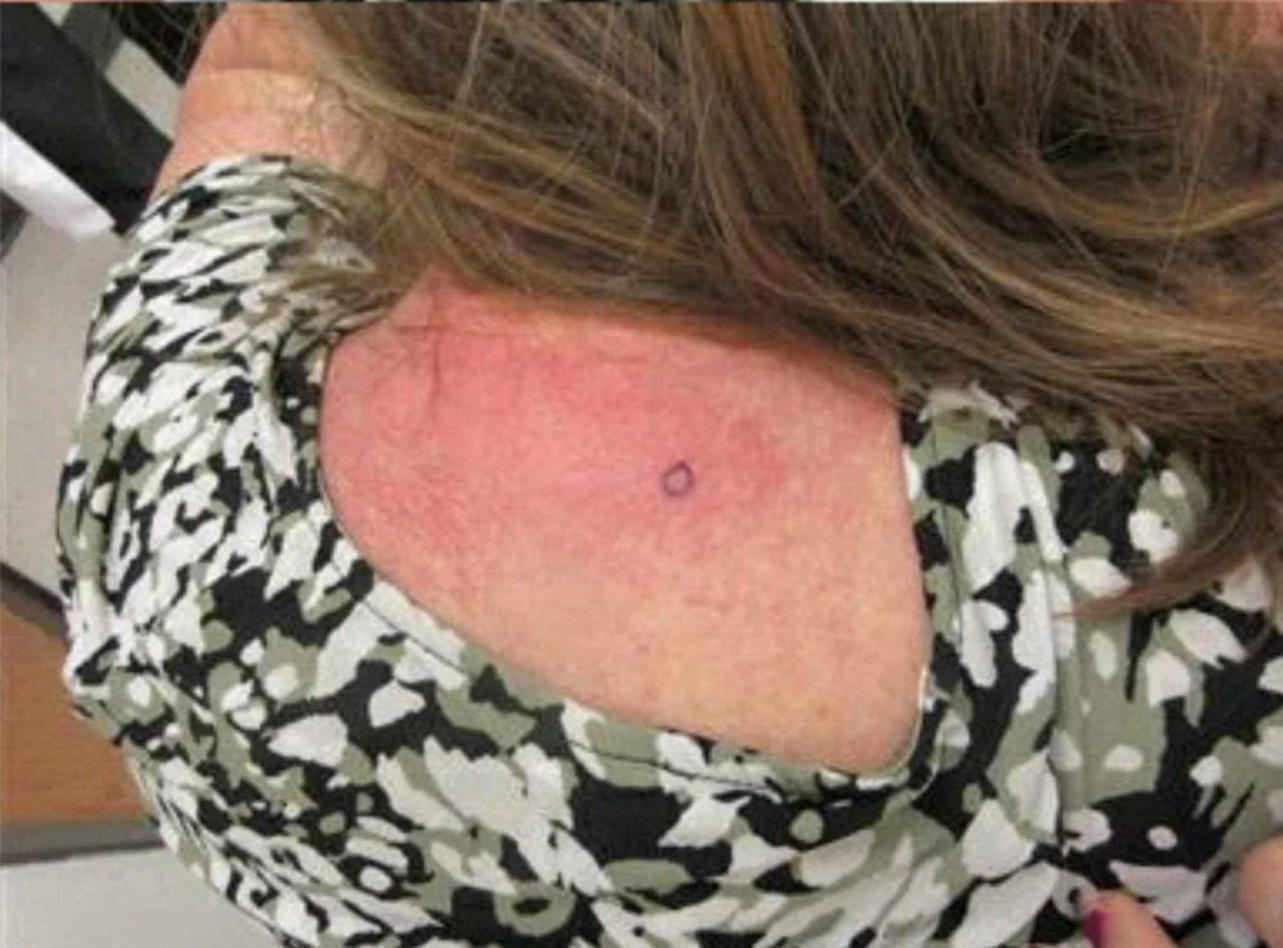
Picture of the rash over the patient’s upper back showing also the biopsy site (blue circle).

**Figure 5:**
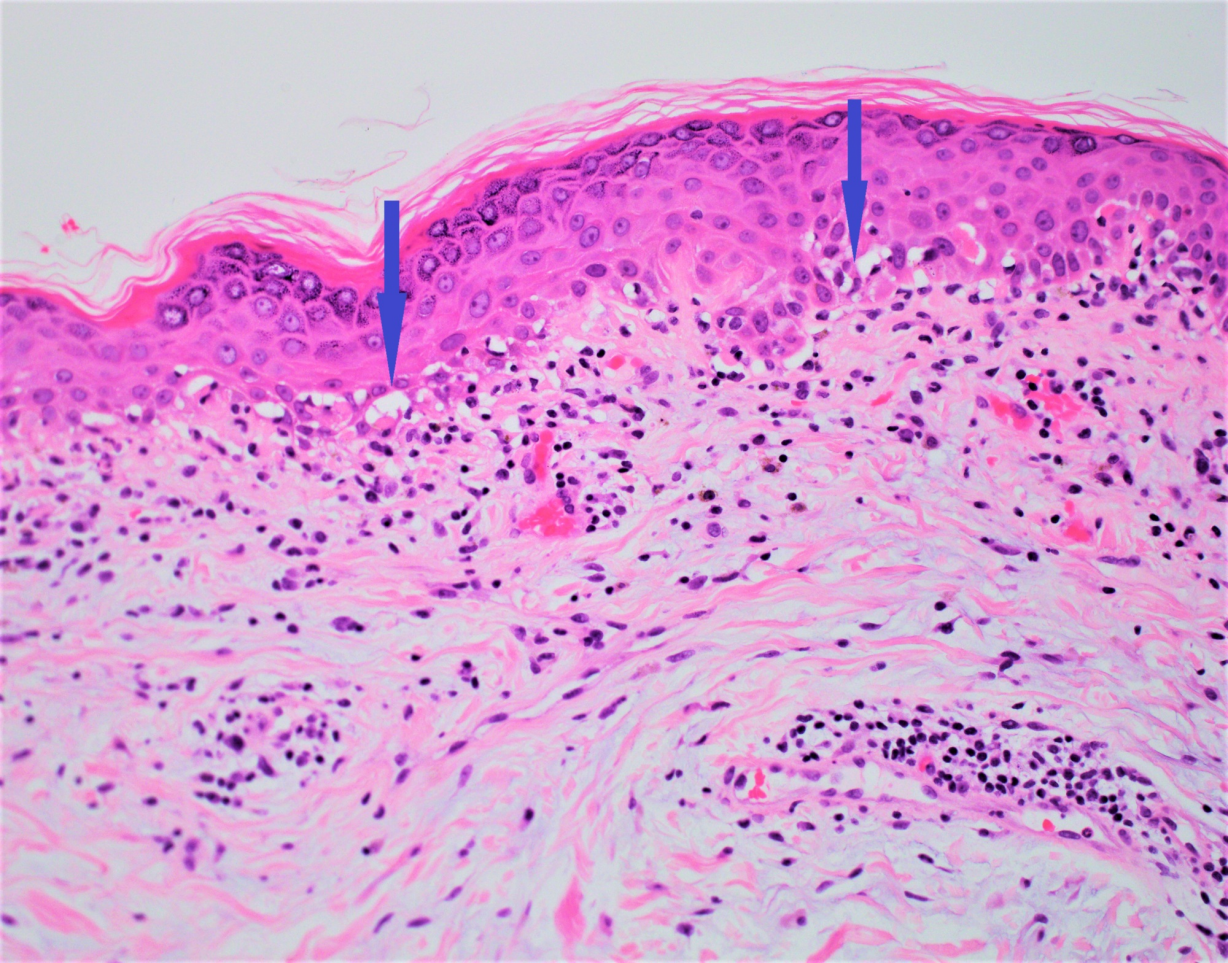
Skin biopsy showing vacuolar interface dermatitis, a classic skin biopsy finding in dermatomyositis.

The patient was referred to Dermatology. A punch biopsy of the skin was performed which revealed dermatomyositis (Figures 4, 5).

**Figure 6:**
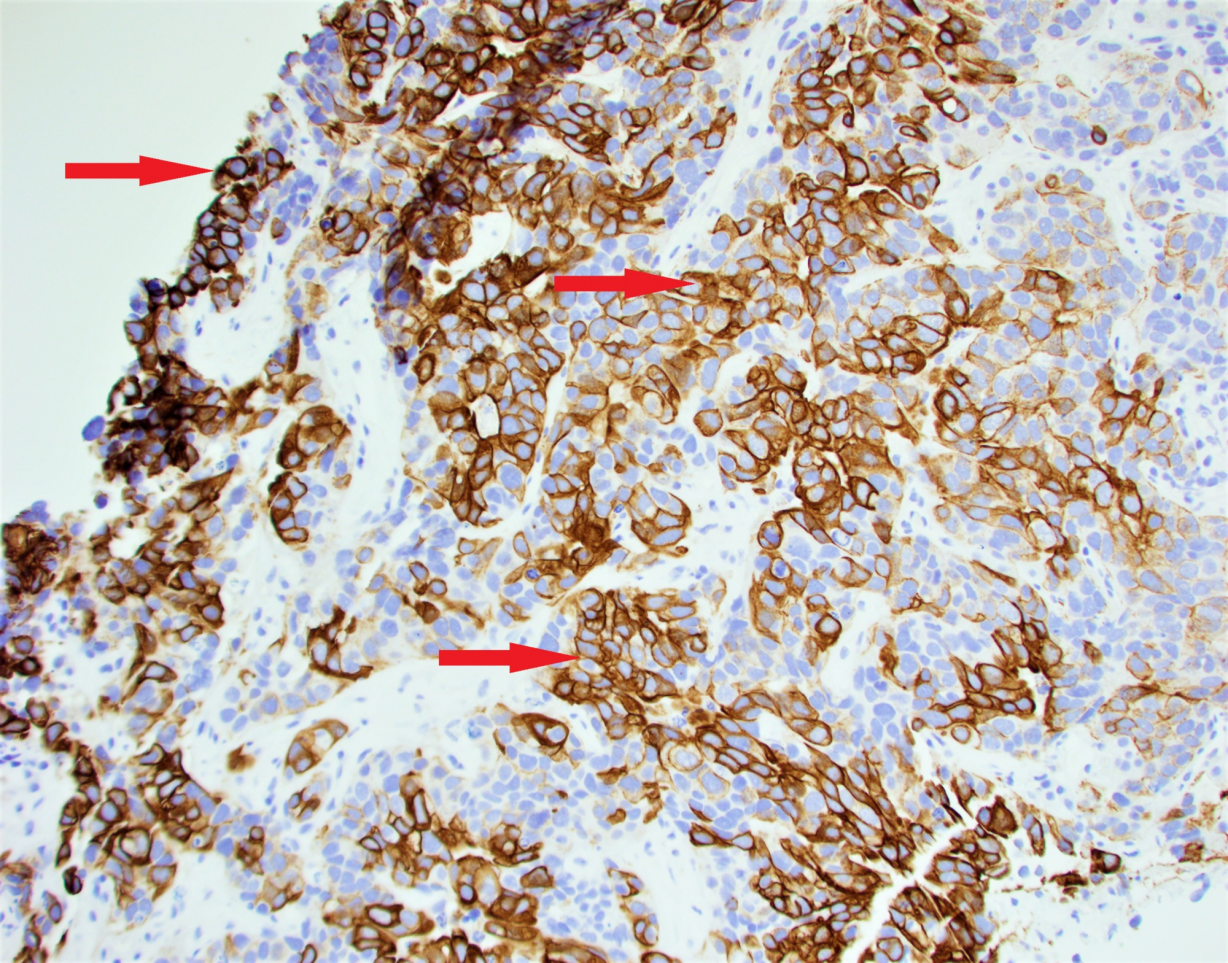
Liver biopsy showing the tumor cells positive for cytokeratin 7 immunostaining.

**Figure 7:**
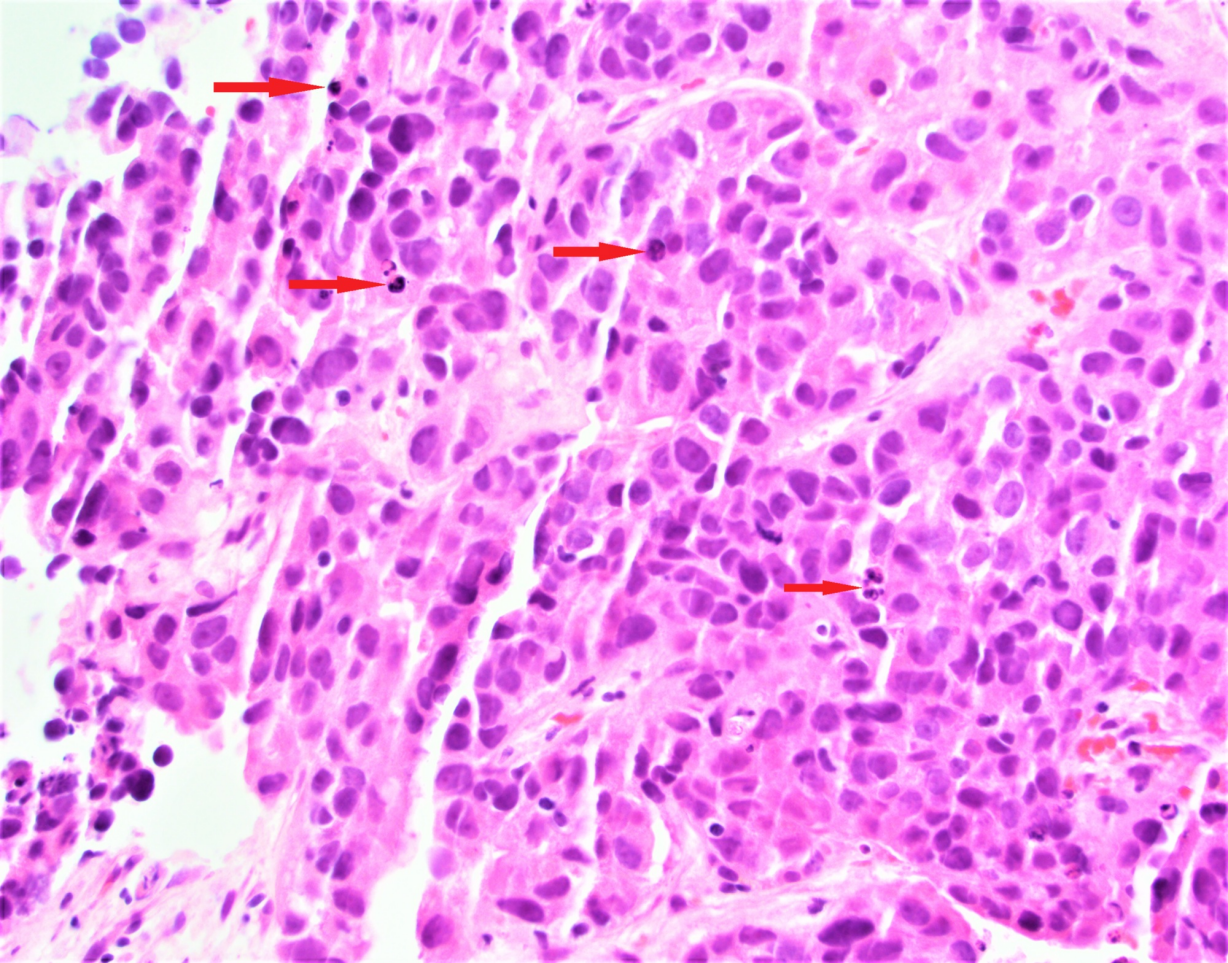
Hematoxylin and Eosin stain of the liver biopsy showing poorly differentiated cells suggestive of malignancy.

The patient was evaluated by the Hematology/Oncology service, where an ultrasound-guided liver biopsy was performed. Biopsy results were suggestive of metastatic adenocarcinoma, and along with elevated CA125 tumor marker, a diagnosis of stage IV metastatic Mullerian adenocarcinoma was made (Figures 6, 7).

A chemotherapy regimen was initiated after a detailed discussion with the patient about the possible risks and benefits. Our patient continues to take chemotherapy and has a marked improvement in the rash.

## Discussion

Dermatomyositis is an idiopathic inflammatory myopathy that is characterized by an inflammatory infiltrate affecting muscle and skin mainly [2]. Dermatomyositis may present with a heliotrope rash, shawl sign, periungual telangiectasias, Gottron’s papules, Gottron’s sign, and proximal muscle weakness. It tends to follow a bimodal distribution between the ages of 5-15 and 45-64 years. Our patient presented at a later age, at the age of 69 years. The progression in our patient was also rapid, within 4-5 weeks, as compared to a 3-6-month gradual onset typically noted. The distribution of the rash was mainly on the anterior chest wall. Also, the patient had skin lesions but did not have muscular weakness - a condition referred to as amyotrophic dermatomyositis.

Bohan and Peter laid down the criteria for the diagnosis of dermatomyositis which included symmetric proximal muscle weakness, elevation of serum skeletal muscle enzymes, characteristic electromyographic pattern, muscle biopsy evidence of myositis and a typical skin rash of dermatomyositis. According to their criteria, dermatomyositis is considered as a definite diagnosis if a skin rash is associated with any three of the above-mentioned criteria [3].

The initial evaluation of dermatomyositis typically involves serum creatinase, liver function tests, lactate dehydrogenase, and aldolase [2]. Our patient had elevated LDH, but other labs were within normal limits. A skin biopsy confirmed the diagnosis. Studies have shown that about 25-42% of patients with Dermatomyositis have an underlying malignancy, especially if the patient is over 45 years of age, and the association becomes stronger if above 60 years of age [4,5]. It may hence be considered as a paraneoplastic syndrome. The associations are stronger with ovarian, lung, gastrointestinal carcinomas. A handful of intracranial neoplasms have also been reported. Dermatomyositis may present either before or after a diagnosis of cancer is made. In our patient, she was still being evaluated for possible carcinoma.

In cases involving proximal muscle weakness, the goal of therapy is to increase muscle strength to enable patients to perform daily activities. Immunosuppression with systemic steroids remains the main therapeutic modality. The doses are 0.5-1.0 mg/kg per day orally or high dose pulse of 1000 mg IV methylprednisolone for 3-5 days [6]. Several case reports have been published stating the influence of symptom improvement after treating the underlying malignancy [7]. Recurrences of dermatomyositis may occur even after treatment. These patients should thus have follow-ups to monitor their muscular weakness.

As there is a great association of dermatomyositis with malignancy, this case points out an important clinical practice that should include a screening of patients for malignancy, especially if the presentation is after 45 years of age. The case also highlights the fact that dermatomyositis in the geriatric age group is a possibility. Up to 20 percent of dermatomyositis can occur in patients over 65 years of age [8].

## Conclusions

Dermatomyositis presents with a variety of manifestations. Our patient presented with skin manifestation in the sun exposed areas (shawl sign), which could easily be overlooked as sunburn when presenting as an isolated manifestation. Muscular manifestation at this age could be confused with aging or statin use and overlooked. Given the rarity of cases, a standard screening guideline is unlikely. A clinician should use clinical gestalt and consider dermatomyositis as a differential diagnosis for skin rash, especially in the patients who are at high risk or have an established diagnosis of malignancy.
